# A Case of 25 Inappropriate Automatic Implantable Cardioverter Defibrillator Shocks and 22 Episodes of Antitachycardia Pacing

**DOI:** 10.7759/cureus.35634

**Published:** 2023-03-01

**Authors:** Nathan Morrison, Navya Voleti, Michael Cannizzaro

**Affiliations:** 1 Emergency Medicine, Penn State Health Milton S. Hershey Medical Center, Hershey, USA; 2 Internal Medicine, Penn State Health Milton S. Hershey Medical Center, Hershey, USA

**Keywords:** lead complication, inappropriate shock, automated implantable cardiac defibrillator (aicd), a case report, emergency medicine

## Abstract

An implantable cardioverter defibrillator (ICD) can save lives from fatal tachyarrhythmias. In rare cases, these devices can fail or malfunction. We present a case of a patient that suffered from 25 inappropriate shocks and 22 episodes of antitachycardia pacing (ATP), secondary to a probable non-traumatic dual lead fracture. One episode of ATP induced an R-on-T phenomenon, causing monomorphic ventricular tachycardia in the patient. The inappropriately functioning ICD also required two magnets to be placed on the patient’s chest in the emergency department to convert the device to an asynchronous mode. An unexpected case of this magnitude and in such a brief timeframe has not been reported in prior ICD studies.

## Introduction

An implantable cardioverter defibrillator (ICD) can save the life of a patient when firing appropriately. These devices are programmed to shock patients experiencing ventricular tachyarrhythmias such as ventricular tachycardia (VT) or ventricular fibrillation (VF) that would not terminate spontaneously, or by antitachycardia pacing (ATP). Although life-saving, the shocks experienced by the patients cause significant morbidity [1]. Inappropriate shocks by pacemakers can be caused by rhythm misdiagnosis, supraventricular tachycardia, atrial fibrillation, abnormal sensing, sinus tachycardia, and other unclassified causes. The Journal of the American College of Cardiology reported that approximately 18% of patients experience an inappropriate shock over a five-year follow-up period [2,3]. Five or more inappropriate shocks result in a hazard ratio of 3.7 for all-cause mortality [2]. The Second Multicenter Automated Defibrillator Implantation Trial (MADIT II) disclosed that only nine out of 719 patients (1.3%) of patients followed in the study were recorded to have >5 inappropriate shocks. The greatest number of inappropriate shocks recorded in the study was 16 episodes for one patient, with a reported mean of 2.2 ± 2.5 episodes over an 18-month follow-up period [4].

Lead fractures can lead to oversensing and inappropriate application of electric therapy, although these lead fractures rarely occur at a rate of 0.58% per year of modern devices [5]. An ICD lead fracture can occur due to trauma, physical exertion, or mechanical manipulation, also known as Twiddler’s syndrome. These device leads do not typically spontaneously fracture. Of note, ATP was developed as a method to pace-terminate certain ventricular arrhythmias and avoid painful shocks. In this case, we present a patient that experienced 25 inappropriate shocks and 22 ATP episodes, secondary to spontaneous dual lead fracture, occurring prior to presentation. The patient suffered from inappropriate ATP causing an R-on-T phenomenon, leading to monomorphic VT in the emergency department, and required two magnets placed simultaneously to change the device to an asynchronous pacing mode, while suspending antitachycardia therapies. The patient appeared to be 100% pacer dependent while on the monitor but was not actually being paced.

## Case presentation

A male in his 70s was brought to the ED by emergency medical services for “several shocks by his ICD.” On initial evaluation, he was awake and alert, with stable vital signs, and reportedly was sitting in a chair when the shocking episodes began. During transport, the patient was given two sublingual nitroglycerin and placed on a cardiac monitor with no abnormal rhythms noted by EMS. The patient had a past medical history of heart failure with an ejection fraction of 30%, atrial flutter, ventricular tachycardia (VT), hypertension, type 2 diabetes, chronic kidney disease, and Parkinsonism. He was on amiodarone for his history of arrhythmias and had a dual chamber automatic implantable cardioverter defibrillator (AICD) placement.

When the patient arrived at the ED, he was hemodynamically stable, nontoxic-appearing, and mentating well. Shortly after being placed in a room, the patient experienced another shock from his ICD, assumed to be inappropriate, with the cardiac monitor displaying a normal sinus rhythm at a normal rate with intermittent pacing. A bedside point of care i-STAT was obtained which showed no acute chemistry, electrolyte, or blood gas abnormalities. His initial chest X-ray showed no significant abnormal ICD or lead issues. The patient denied any chest pain, shortness of breath, headache, lightheadedness, or dizziness, only becoming uncomfortable during episodes of ICD shocks. Shortly after arrival, the patient converted into a wide-complex tachycardia (monomorphic VT) at an approximate rate of 140 beats per minute. During this rhythm, the patient became hypotensive with mean arterial pressure (MAP) in the 50s, but he maintained normal mentation and was therefore administered one liter of intravenous crystalloid fluids on a pressure infusion bag with 150 mg of amiodarone. The patient was then shocked by his ICD after approximately two minutes in the stable monomorphic VT rhythm, after which he converted to a normal sinus rhythm. The patient continued to receive several inappropriate shocks in the ED and was administered 50 mcg of fentanyl for pain control. During the initial resuscitation, cardiology was emergently consulted and presented at the bedside. A magnet was placed on the pacer to convert the device to an asynchronous mode, however, the patient continued to receive ICD shocks. A second magnet was placed on top of the first, without adjusting the location, which appeared to convert the ICD to an asynchronous mode. The pacemaker device was then turned off by the cardiology team.

**Figure 1:**
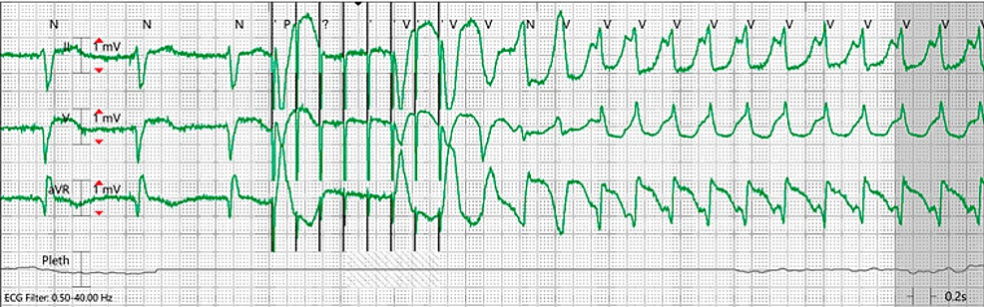
R-on-T phenomenon due to inappropriate antitachycardia pacing leading to monomorphic ventricular tachycardia

**Figure 2:**
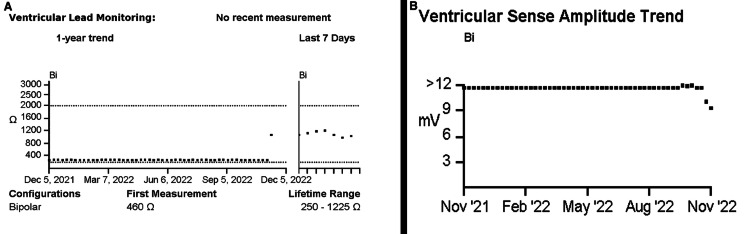
Device interrogation of ventricular lead monitoring and ventricular sense amplitude trend A: Sudden increase in ventricular lead impedance is observed B: Decrease in ventricular lead sensing amplitude is observed The device interrogation showed a sudden increase in ventricular lead impedance and a decrease in ventricular lead sensing amplitude, supporting a spontaneous lead fracture.

The pacemaker interrogation indicated inappropriate tachyarrhythmia therapies including 25 shocks and 22 ATP episodes the morning he presented to the ED. Additionally, there was an episode of monomorphic VT induced by inappropriate ATP in the ED (Figure 1). The RV lead impedance rose to 950 ohms from 280 ohms and the RA impedance increased from 290 ohms to 310 ohms (Figure 2 A & B). Historically, the patient was atrial paced at 7%, ventricular paced at 1.3%, although appeared to be briefly pacing 100% in both chambers. Further investigation revealed laboratory studies significant for an elevated N-terminal pro-b-type natriuretic peptide (NT-proBNP) of 5999 pg/mL, hypocalcemia (ionized calcium 1.05 mmol/L), increased serum creatinine of 1.88 mg/dL (baseline 1.3 mg/dL - 1.5 mg/dL) and elevated TSH of 4.97 uIU/mL, but otherwise unremarkable. The patient was emergently taken to the electrophysiology laboratory and a new RV ICD lead and generator were placed. On discharge, the patient’s rhythm was in atrial fibrillation and flutter.

## Discussion

An emergency medicine (EM) physician may be faced with a malfunctioning pacemaker in their career. In this case, the patient experienced 25 inappropriate shocks and 22 episodes of ATP, which had not been quantified or reported previously [4,6]. The 2016 Model of the Clinical Practice of Emergency Medicine was developed in collaboration between six leading organizations in emergency medicine. This cohort determined that ICDs are a core component for EM physicians to master [7]. This case highlights several unique and important features for all EM physicians. Spontaneous non-traumatic AICD dual lead fractures may occur prior to presentation (as seen above in Figure 2). Inappropriate ATP can cause an R-on-T phenomenon leading to monomorphic VT in the ED and must be recognized (as seen above in Figure 1). The need for a second magnet to convert an AICD to an asynchronous pacing mode and suspend antitachycardia therapies during initial stabilization may be warranted, hence knowledge of the location of the magnets is important. In this case, the first magnet was not adjusted before the placement of the second magnet. The second magnet may have increased the magnetic performance of the Reed switch in the patient's AICD. Lastly, a discrepancy in rhythms read by the AICD and telemetry may exist. For instance, in this case, normal sinus rhythm was on the monitor while the AICD was interpreting monomorphic VT.

## Conclusions

In our case, all 25 inappropriate shocks and 22 episodes of ATP occurred prior to and during stabilization in the ED, presumed to be caused by a spontaneous dual lead fracture. As defined by the Antiarrhythmics Versus Implantable Defibrillators (AVID) study, an inappropriate shock occurring within five minutes was not counted for analysis. By this definition, the patient in our case suffered 11 inappropriate shocks, and 12 episodes of ATP when he arrived at the ED. To our knowledge, the magnitude of this patient’s inappropriate shocks has not been declared in prior literature, in such an acute time period.
